# Luteinizing Hormone Effect on Luteal Cells Is Dependent on the Corpus Luteum Stage in Felids

**DOI:** 10.3390/ani11010179

**Published:** 2021-01-14

**Authors:** Michał M. Hryciuk, Katarina Jewgenow, Beate C. Braun

**Affiliations:** Leibniz Institute for Zoo and Wildlife Research, Department of Reproduction Biology, 10315 Berlin, Germany; jewgenow@izw-berlin.de (K.J.); braun@izw-berlin.de (B.C.B.)

**Keywords:** felids, luteal cells, steroidogenic activity, luteinizing hormone, domestic cat

## Abstract

**Simple Summary:**

The corpus luteum is a transient endocrine gland on the mammalian ovary, and its main function is to produce progesterone. Knowledge about the corpus luteum in felids is very limited and luteolytic and luteotrophic factors which regulate its maintenance and regression are not extensively studied. Information about corpus luteum function is needed to understand breeding strategies and to successfully implement assisted reproductive techniques for felids, of which most of the species are threatened. The aim of this study was to reveal the effect of luteinizing hormone on cultured luteal cells from corpora lutea obtained from selected felids and to investigate the protein expression of steroidogenic enzyme 3β-hydroxysteroid dehydrogenase by immunohistology.

**Abstract:**

The objective of this study was to investigate the effect of luteinizing hormone (LH) on steroidogenic luteal cells obtained from corpora lutea (CL) of the domestic cat and selected wild felids. Luteal cells were isolated enzymatically from CL at different developmental stages and cultured for two days in the presence and absence of 100 ng/mL LH, respectively. Functionality was assessed by progesterone (P4) accumulation in cell culture media determined by ELISA. In addition, steroidogenic function was confirmed using immunohistochemistry for 3β-hydroxysteroid dehydrogenase (HSD3B). The enzymatic method allowed for the isolation of mostly small luteal cells in all investigated felids. Treatment with LH resulted in an increase in P4 secretion of cultured luteal cells obtained from CL in the formation stage (African lion) and development/maintenance stage (domestic cat (*p* < 0.05), Javan leopard), whereas luteal cells from more advanced stages of luteal development (regression) responded moderately or not at all to LH stimulation (domestic cat, Asiatic golden cat, Asiatic lion). The protein signal for HSD3B on CL was visible until development/maintenance. In conclusion, this study shows that LH promotes P4 production in luteal cells only until the onset of regression, when morphological signs are visible on the CL of felids and HSD3B is no longer detectable.

## 1. Introduction

There are 38 wild felids listed on the IUCN Red List, and 25 of them are classified as near threatened, vulnerable or endangered (https://www.iucnredlist.org). The main causes of felid population decline are due to habitat loss and fragmentation, human-wildlife conflicts, the illegal pet trade and traditional medicine [1]. To protect species from extinction, ex situ and in situ conservation programs have been established, but success also depends on knowledge on the reproduction cycles of felids. Among felids, there are species whose reproductive biology have never been described [2]. For only 24 species, some hormonal reproduction patterns are known currently [3]. Therefore, there is a critical need for more information about reproductive biology of felids to improve natural breeding or for the application in assisted reproductive techniques (ART) [4,5].

It is known that in most felids, ovulation is induced by copulation or some other form of physical or social interaction [4,6], but some of these species also ovulate spontaneously, in particular when a mating partner is not available [4]. Ovulation is followed by the formation of corpus luteum (CL), which is a temporary gland on the ovaries. Its main function is the production of progesterone (P4) and maintaining pregnancy. In most studied species, the CL is composed of steroidogenic small (SLC) and large (LLC) luteal cells and non-steroidogenic cells such as fibroblasts, endothelial cells, pericytes and cells originating from the bloodstream [7]. During its lifespan, four main stages can be distinguished—formation (f), development/maintenance (dm), early regression (er) and late regression (lr) [8].

Knowledge on CL in felids is limited mainly to domestic cats and lynxes. The functional lifespan of CL in the domestic cat based on serum progesterone level should last around 40 days in case of non-pregnant cycle (pseudopregnancy) and around 65 days during pregnancy [9,10]. In non-domestic felids, the pregnancy length depends on the species and may last from 56 to 127 days [2]. The non-pregnant luteal cycle usually takes around half of the gestation period [2]. The luteal cycle usually ends with a full structural regression to corpora albicans. In the domestic cat, regressing CL or corpora albicans remain visible on the ovary for up to eight months [11]. Contrary, the CL of lynxes are characterized by a unique lifecycle. Formed after ovulation, CL do not regress after pregnancy (or pseudopregnancy) and lactation but transform into persistent CL, which remains hormonally active for a period of at least two years [12].

Luteinizing hormone (LH) is a luteotrophic hormone whose action is crucial for the formation of CL [13]. This hormone is responsible for initiating differentiation of granulosa and theca cells to large luteal cells (LLC) and small luteal cells (SLC), respectively [14,15]. In the domestic cat, during estrus, LH quickly increases for a short period; then, its concentration decreases and oscillates to around 5 ng/mL [16,17]. Furthermore, during estrus, two major peaks are detectable [18].

The influence of LH on CL of felids is not extensively studied. Functional testing on CL survival in any felid is limited due to its invasive character, regulations on animal experimentation and side effects on animals [19]. In addition, many felids are threatened which excludes any experimental access to the animal. Therefore, the only rational solution for functional testing is in vitro studies on cultured luteal cells.

The aim of this study was to investigate an effect of LH (100 ng/mL) on luteal cells from the domestic cat and selected wild felids during two days of culture. In addition, the expression of 3β-hydroxysteroid dehydrogenase (HSD3B) was confirmed by immunohistology on CL tissues. The experiments we performed on CL from the formation, development/maintenance and early and late regression stages. The enzymatic isolation method and culture conditions previously described for domestic cats were applied [20]. The presented studies were conducted to contribute to knowledge about CL of felids.

## 2. Materials and Methods

This study was approved by the Internal Committee for Ethics and Animal Welfare of the Leibniz Institute for Zoo and Wildlife Research (IZW) (2017-02-02). Ovarian samples from domestic cats were obtained from routinely ovariectomized queens in an animal shelter in Berlin. The reason for the ovariectomies was not related to the purpose of this experiment. Analyzed samples from wild felids were obtained from captive animals during necropsy at the wildlife pathology lab of the IZW or were isolated after euthanasia for management or health reasons in European zoos and were transported to the IZW under the Felid Gametes Rescue Project [21]. Detailed information about individuals from wild felids is listed in Table 1.

All chemicals used in the experiments were purchased from Merck KGaA (Darmstadt, Germany) unless otherwise stated.

### 2.1. Samples Transport and Isolation of CL

The samples from wild felids were transported to the lab in plastic tubes filled with saline solution within 24 h after extirpation of gonads. During transportation, the tubes were cooled by ice packs. The samples from domestic cats were delivered to the laboratory within around five hours after surgery. Immediately upon receiving, ovaries of felids were washed in Dulbecco’s Phosphate Buffered Saline (DPBS) and CLs were dissected, washed in DPBS, trimmed to remove connective tissue and washed once again. From each individual, a small piece of one CL was cut out and incubated in Bouin solution for standard histology by hematoxylin and eosin staining and for immunohistochemistry (see below).

### 2.2. Isolation of Luteal Cells

Enzymatic isolation of small luteal cells from the domestic cat has been described previously [20] and was used without modifications in this study. Briefly, CLs were chopped on a petri dish with culture medium (HAM’s F12 and MEM Eagle medium 1:1 (v:v) supplemented with 5% of fetal bovine serum (FBS) and 0.055 mg/mL gentamicin). The luteal tissue pieces were then transferred onto a 40 µm cell strainer, immersed in culture medium supplemented with collagenase 0.1% (type I and II, SERVA Electrophoresis GmbH) and 0.005% DNase. The tissue pieces were digested at 39 °C for 55 min and were thereafter gently smashed through the cell strainer. This cell suspension was washed by centrifugation (7 min, 1000× *g*) and the cell pellet was re-suspended in a 2 mL culture medium. The cell suspension was laid on a 40% percoll layer in a test tube and centrifuged (7 min, 1000× *g*). Afterwards, luteal cells were collected from the interphase and were washed again by centrifugation (4 min, 500× *g*). Finally, the cells were re-suspended in fresh culture medium and the cell count was determined in a hemocytometer. Viability staining of the cells was not performed due to low number of obtained cells and its unique character. All isolated cells were used to increase number of technical repeats within treatment groups.

### 2.3. Cell Culture Conditions and Cell Diameter Evaluation

The obtained luteal cell suspension was divided into a control group (culture medium) and an LH treatment group. The cell concentration was brought to 200,000 cells per mL by diluting the cells with the respective culture medium. The final concentration of LH (from human pituitary) in the treatment group was 100 ng/mL. Cells (30,000 cells per 150 µL) were seeded into 96-well Tissue Culture Plates (Sarstedt AG & Co KG, Nümbrecht, Germany) previously coated with 15 µL of 0.02% Collagen R (Serva Electrophoresis GmbH, Heidelberg, Germany) in DPBS to improve cells attachment. The total number of technical and biological replicates for each species is shown in Table 1. Cells were cultured for two days at 39 °C, 5% CO_2_. The cell culture medium was changed each day by replacing 130 µL medium with a freshly prepared one. The collected medium was stored at −20 °C until hormone extraction. Each day, the cells were microscopically analyzed under the Axiovert 200 M microscope (Carl Zeiss, Oberkochen, Germany), which was equipped with a ProgRes^®^ C3 camera (JENOPTIK Optical Systems GmbH, Berlin, Germany) that was connected to a computer with the program CapturePro 2.20.01. Photos were taken and archived to measure the cell diameters with the program cell^D (Olympus Soft Imaging Solutions GmbH, Münster, Germany).

### 2.4. 3β-hydroxysteroid dehydrogenase Assay

Identification of steroidogenic luteal cells was performed by determination of enzyme activity of 3β-hydroxysteroid dehydrogenase (HSD3B) in freshly isolated and cultured cells [20]. The assay was performed on day two of culture. Before staining, the cells were fixed in 1% formaldehyde solution at 39 °C for 15 min, followed by washing twice in DPBS and adding the staining/control solution (1.5 mM nicotinamide adenine dinucleotide, 0.25 mM nitrotetrazolium blue chloride, 0.2 mM pregnenolone, 2 mM ethylenediaminetetraacetic acid, 0.1% BSA). Cells were left in a staining/control solution for overnight incubation at 39 °C. On the following day, cells were washed twice in DPBS and the staining intensity within cells was documented by microphotography. For control, activity of 3β-hydroxysteroid dehydrogenase was blocked by 2 mM trilostane as described before [20].

### 2.5. P4 Extraction and ELISA Measurements

Hormone extraction and measurement was done as described before [20]. Hormones were extracted from 100 µL culture media supernatant. Extracts were dissolved in 200 µl 40% methanol and were stored at −20 °C until P4 measurements. P4 analyses were carried out with an in-house microtiter plate enzyme immunoassay as described earlier [22] using a commercial P4 antibody (Sigma P1922, raised in rats to P4) and 4-pregnen-3,20-dione-3-CMO-peroxidase label. The cross-reactivity to other steroids were as follows: 4-pregnen-3,20-dione (P4), 100%; 5a-pregnan-3,20-dione, 31%; 5a-pregnan-3b-ol-20-one, 18%; 5-pregnen-3b-ol-20-one, 12%; 4-pregnen-3aol-20-one, 4.2%; <0.1% for 5b-pregnan-3a,20adiol, 4-pregnen-20a-ol-3-one, 5b-pregnan-3a-ol-20-one, 5a-pregnan-20a-ol-3-one, 5a-pregnan-3a,20 a-diol, 5a-pregnan-3b,20a-diol, testosterone, estradiol and cortisol. Samples were partly diluted with 40% methanol and were measured in duplicates. Parallelism of diluted samples and the linearity of the method were confirmed. Inter- and intra-assay coefficient results for the two biological samples were 11.0% and 4.8% and 7.6% and 2.8%, respectively. Extraction recovery for the control sample was 88.9–99.7%. Results for samples after two days in culture describe the values with the subtracted amount of remaining progesterone produced during the first day of culture period. This correction was done on a per-well basis.

### 2.6. Immunohistochemistry and Evaluation of Immunoreactivity

Immunohistochemistry (IHC) was performed as described previously in Braun et al. [23]. Briefly, CL tissue pieces were fixed in Bouin’s solution, embedded in paraffin, sliced to 3 µm thicknesses and mounted on microscope slides (Superfrost Plus, Thermo Scientific). The slides were then deparaffinized in Roti Histol (Carl Roth GmbH) and rehydrated in decreasing concentrations of ethanol. Afterwards, slides were incubated overnight with diluted primary antibody (Table 2). Control staining was incubated with blocking solution; furthermore, we tested and confirmed that the primary antibodies did not cause an unspecific staining pattern on ovaries (not shown). The next day, the slices were incubated with the appropriate second antibody solution (Table 2) before binding of antibodies was visualized by incubation for 5 min for all samples with diaminobenzidine (DAB) substrate chromogen solution (Dako Deutschland GmbH, Hamburg, Germany). Sections were counterstained with hematoxylin dehydrated in increasing concentrations of ethanol, and covered with mounting medium and coverslips. Stained CL were observed under inverted microscope (IX81, Olympus Deutschland GmbH, Hamburg, Germany) with a 40× objective. Pictures of the CL sections from different stages and species were taken with a DP72 camera (Olympus) and CellSens imaging software (Olympus) with the same settings to provide identical conditions. HSD3B was chosen as a representative steroidogenic enzyme, since we could show in a former study that the HSD3B protein signals (detected by Western Blot with the same antibody) reflected the intraluteal P4 values in domestic cat CL [24]. Results and conclusions for immunohistochemistry are based on staining for three individuals at formation, development/maintenance and early and late regression stages for domestic cats and single individual wild felids.

### 2.7. Assessment of CL Stages in Wild Felids

**Figure 1:**
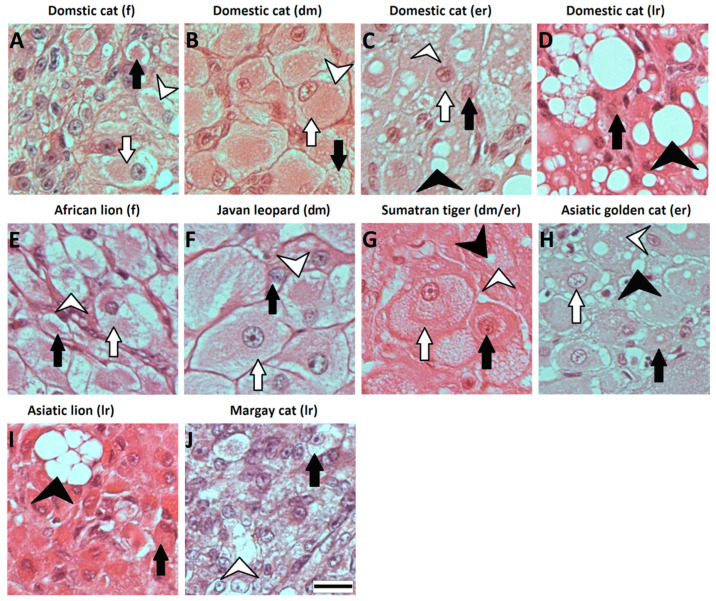
Hematoxylin and eosin staining of corpus luteum (CL) from domestic cat (**A**–**D**) and wild felid (**E**–**J**) species at different developmental stages. (**A**) CL from the domestic cat in the formation stage—luteal cells have different sizes and shapes, from elongated to round or polyhedral. Small vacuoles are present in the cell cytoplasm; (**B**) CL from the domestic cat in the development/maintenance stage—luteal cells are fully grown, have a polyhedral shape and contain high degree of small vacuoles; (**C**) CL from the domestic cat in the early regression stage—luteal cells contain small and large vacuoles and their shape may be partly deformed; (**D**) CL from the domestic cat in the late regression stage—luteal cells are deformed because of the presence of high numbers of large vacuoles; (**E**) CL from the African lion in the formation stage—luteal cells have different sizes and small vacuoles; (**F**) CL from the Javan leopard in the development/maintenance stage—luteal cells are large or medium-sized and have polyhedral shape, indicating a development rather than a maintenance stage; (**G**) CL from the Sumatran tiger in the development/maintenance–early regression stage—luteal cells have a degree of small vacuoles with single large vacuoles in some of the cells; (**H**) CL from the Asiatic golden cat in the early regression stage—luteal cells have a round or polyhedral shape with large and small vacuoles; (**I**) CL from the Asiatic lion in the late regression stage—single small (SLC) or large (LLC) luteal cells with a round shape are visible, but most of the cells are small and deformed. Rare large vacuoles are visible; (**J**) CL from Margay cat in the late regression stage—single SLC or LLC with an oval shape are visible while other cells are densely packed. A black arrow indicates SLC; a white arrow indicates LLC; a white arrow head indicates small vacuoles; a black arrow head indicates large vacuoles. f—formation, dm—development/maintenance, er—early regression, lr—late regression. Scale bar is equal to 20 µm.

The assessment based on previously described characteristic for the domestic cat [8] was combined with morphological features of the gland, such as the presence of an ovulation scar, CL size and its location on the ovary. Microphotographs of CLs at different developmental stages from domestic cats were compiled together with microphotographs of CLs from different wild felids for comparison (Figure 1). The CLs of the African lion were not fully formed and had signs of recent ovulation. The gland was composed of steroidogenic luteal cells of different sizes and shapes from elongated to round or polyhedral (Figure 1E). The cells had a moderate number of small vacuoles localized mainly in the cells’ periphery and the CL showed signs of neovascularization. All of those features were similar to CLs from domestic cats at the formation stage (Figure 1A). The sample from the Javan leopard had similarities to CL at development/maintenance of the domestic cat (Figure 1B) because most of the cells were large and had a polyhedral shape, and vacuoles were distributed through the cytoplasm, but some elongated cells were also found (Figure 1F). Asiatic golden cat CL was characterized by oval or round steroidogenic luteal cells with two types of vacuoles; small ones were distributed in a cell cytoplasm and large ones were found only in some of the luteal cells (Figure 1H). The presence of the second vacuole type allowed us to classify the CL to the early regression stage. Classification of CL stages for the Asiatic lion, Margay cat and Sumatran tiger could not be easily assessed. The Sumatran tiger CL was very small in size (a diameter of around 2 mm), which seemed to be comparable with the size of CLs in a late regression stage of domestic cats. Interestingly, the gland was composed of a polyhedral LLC compactly filled with small, moderate or large vacuoles (Figure 1G); therefore, the transition stage between the development/maintenance and early regression stage was suggested. The CL of the Asiatic lion was very small in size. The LLC/SLC were found only on a small area of the gland and they were surrounded by cells whose cytoplasm to nucleus ratio was lower in comparison to the LLC/SLC (Figure 1I). The gland did not show any signs of neovascularization, and it appeared that the regression process was proceeding towards the LLC/SLC area. Despite the lack of many large vacuoles, which are typical for advanced regression in domestic cats (Figure 1D), the CL was classified as late regression. The Margay cat CL was very small-sized and was mainly composed of cells which looked like small luteal cells with a similar nucleus to cytoplasm ration (Figure 1J) and nonsteroidegenic cells. The steroidogenic cells looked like they might have already undergone processes of conversion. Therefore, despite the absence of large vacuoles, the CL were classified to the late regression stage.

### 2.8. Statistical Analysis

Progesterone analysis for the domestic cat describes results from five different individuals in which CL has been classified as being in the development/maintenance stage (*n* = 5) and three individuals in which CL has been classified as being in the early regression stage (*n* = 3) (Table 1). In the statistical analysis, all technical repeats from all biological replicates had been used. A Mann–Whitney Test was used to assess statistical differences between the control and LH test group at the same time point of the cultured cells. The analysis was performed in R (R: A language and environment for statistical computing (2018); R Foundation for Statistical Computing, Vienna, Austria; v. 3.5.0). *p*-values lower than 0.05 were considered as significant. Statistical analysis was not performed for data of wild felids because results represent single individuals (*n* = 1).

The P4 concentration in a medium of cultured luteal cells was shown in a vertical box plot by plotting medians and the 10th, 25th, 75th and 90th percentiles (Sigma.Plot 10.0 Systat software GmbH, Erkrath, Germany).

## 3. Results

### 3.1. Characteristics of Isolated and Cultured Cells

**Figure 2:**
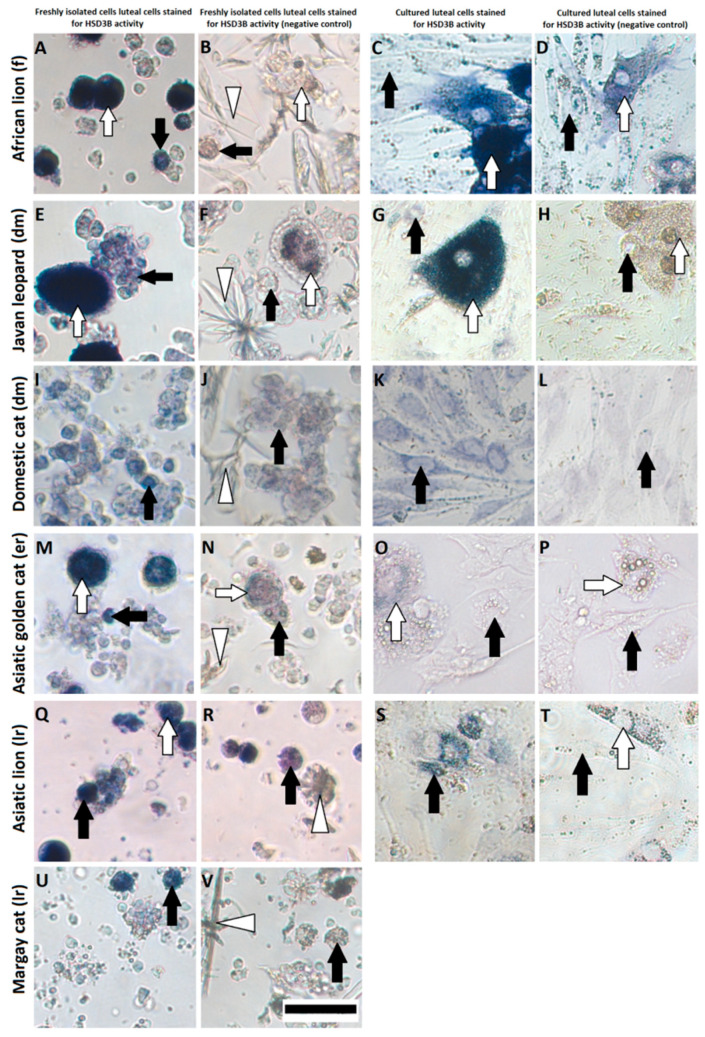
Freshly isolated cells (**A**,**B**,**E**,**F**,**I**,**J**,**M**,**N**,**Q**,**R**,**U**,**V**) and luteal cells cultured for two days (**C**,**D**,**G**,**H**,**K**,**L**,**O**,**P**,**S**,**T**) from the domestic cat (**I**–**L**) and wild felids (**A**–**H**,**M**–**V**) stained for activity of HSD3B. Luteal cells from the Margay cat were isolated but not cultured because of a limited number of obtained cells. Luteal cells with high steroidogenic capacity are stained with a dark blue color. Activity of HSD3B in control groups (**B**,**F**,**J**,**N**,**R**,**V**,**D**,**H**,**L**,**P**,**T**) was blocked by trilostane (2 mM), and the light blue or purple color of these cells come from other NAD+-dependent metabolic reactions. A white arrow indicates LLC; a black arrow indicates SLC; a white triangle indicates a crystal of trilostane. f—formation, dm—development/maintenance, er—early regression, lr—late regression. Scale bar is equal to 50 µm.

Size classification of SLC and LLC followed the previously established description for the domestic cat [20,25]. The SLC were characterized by diameters smaller or equal to 20 µm and LLC by diameter above 20 µm. The average diameter of all isolated cells for each species and percentage of SLC in cell suspension of all isolated cells are presented in Table 3. In all studied species, the average diameter of isolated cells indicated that mainly SLC were isolated; however, histological microphotographs (Figure 1) indicated the presence of a higher numbers of LLC in comparison with SLC in most of studied species. Only the suspension obtained from CL of the Javan leopard contained two very distinctive small and large cells types (Figure 2E), and the relation between SLC and LLC could not be determined here, because the SLC were in clumps, making counting unreliable. Isolated SLC from all analyzed felids had a round shape, and in some of them, small lipid droplets were visible, especially those in which the diameter was greater than 15 µm. The behavior of SLC in the culture was similar to what was described before for the domestic cat, but the LLC of wild felids, in contrast to our previous experiments with the LLC of domestic cats isolated mechanically [20], flattened to the bottom of the culture well.

### 3.2. Identification of Steroidogenic Cells in Freshly Isolated and Cultured Cells

The steroidogenic activity of cells was mirrored by the amount of accumulated formazan within the cells’ cytoplasm (Figure 2). Isolated SLC (usually smaller than 15 µm) of the domestic cat, Javan leopard and Asiatic lion (Figure 2E,I,Q) had a low steroidogenic capacity, which was mirrored by blue or partially blue staining. The SLC in the African lion or Margay cat cultures were characterized by greater number of cells which did not show any steroidogenic activity in comparison to previously mentioned species (Figure 2A,U). All large cells were stained dark blue independently of the stage and species, hinting to a high steroidogenenic capacity (Figure 2A,E,M,Q). During the culture period, LLC also express high steroidogenic capacity, while most of the SLC were characterized by a very low or a lack of HSD3B activity (Figure 2C,G,K,O,S). Only single SLC were able to maintain its steroidogenic capacity to a level comparable with that observed after isolation.

### 3.3. P4 Secretion in Luteal Cell Cultures

**Figure 3:**
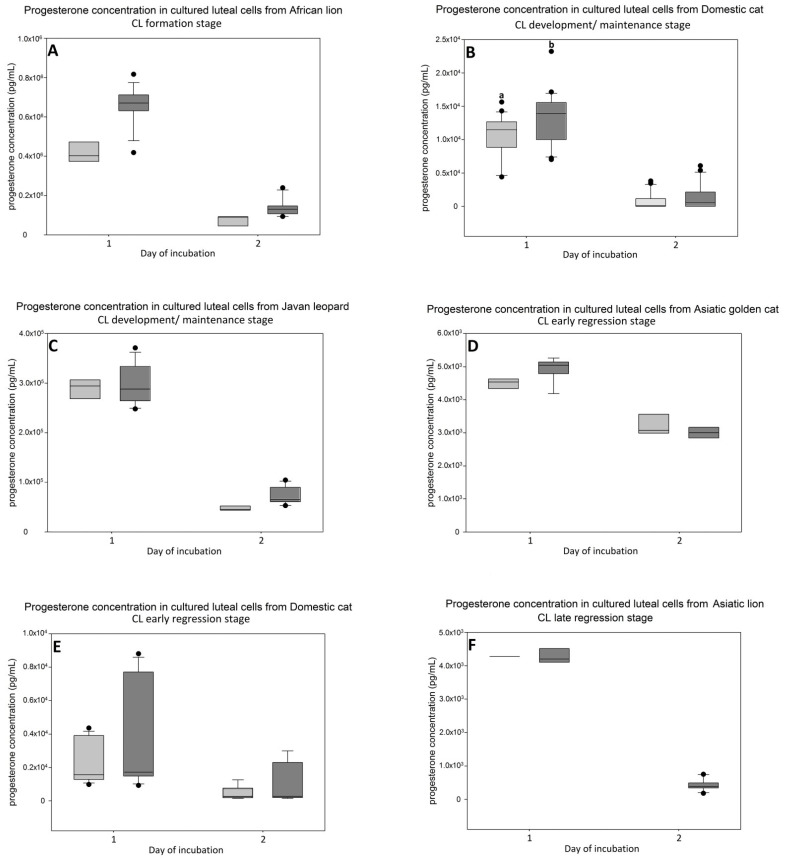
P4 concentration in medium of cultured luteal cells of the domestic cat ((**B**) development/maintenance (*n* = 5), (**E**) early regression (*n* = 3)) and wild felid species at different CL stages ((**A**) African lion (*n* = 1), formation; (**C**) Javan leopard (*n* = 1), development/maintenance; (**D**) Asiatic golden cat (*n* = 1), early regression; (**F**) Asiatic lion (*n* = 1), late regression) showed as box plots of median (horizontal line), 25th and 75th percentiles (lower/upper end of box) and 10th and 90th percentiles (whiskers); black dots indicate outliers. Light grey—control group, grey—LH 100 ng/mL group. Statistical Mann–Whitney was applied only on samples from the domestic cat. Small letters indicate statistically significant different groups (*p* < 0.05) at the same day of incubation. In the cell culture of the Asiatic lion, progesterone concentration in the control group at day 2 was not measured because of limited number of cells.

P4 secretion during the culture period varied greatly between species and developmental stages of CL (Figure 3). For all cell cultures, the highest amount of P4 was measured in medium collected after one day of cell culture. In media of day 2, the P4 concentration decreased. Without LH supplementation, the lowest decrease in P4 concentration was observed in the Asiatic golden cat (CL early regression) (Figure 3D).

For the domestic cat, the concentration of P4 in medium without LH from luteal cells in the development/maintenance stage (*n* = 5) (Figure 3B) was almost five times higher in comparison to cells obtained from early regression (*n* = 3) (Figure 3E). Treatment with LH resulted in a higher average P4 concentration (*p* < 0.05) from the development/maintenance stage, at least during the first day of incubation (Figure 3B), compared to treatment without LH. The average increase in P4 production was 24%.

Treatment with LH resulted in higher measured values (no statistics performed, *n* = 1) for P4 medium concentrations in the African lion (CL formation, Figure 3A, day 1 and 2), Javan leopard (CL development/maintenance, Figure 3C, day 2) and Asiatic golden cat (CL early regression, Figure 3D, day 1). Almost no effect was measured in the CL regression stage of the Asiatic lion (Figure 3F).

### 3.4. Protein Expression of HSD3B by Immunohistology

**Figure 4:**
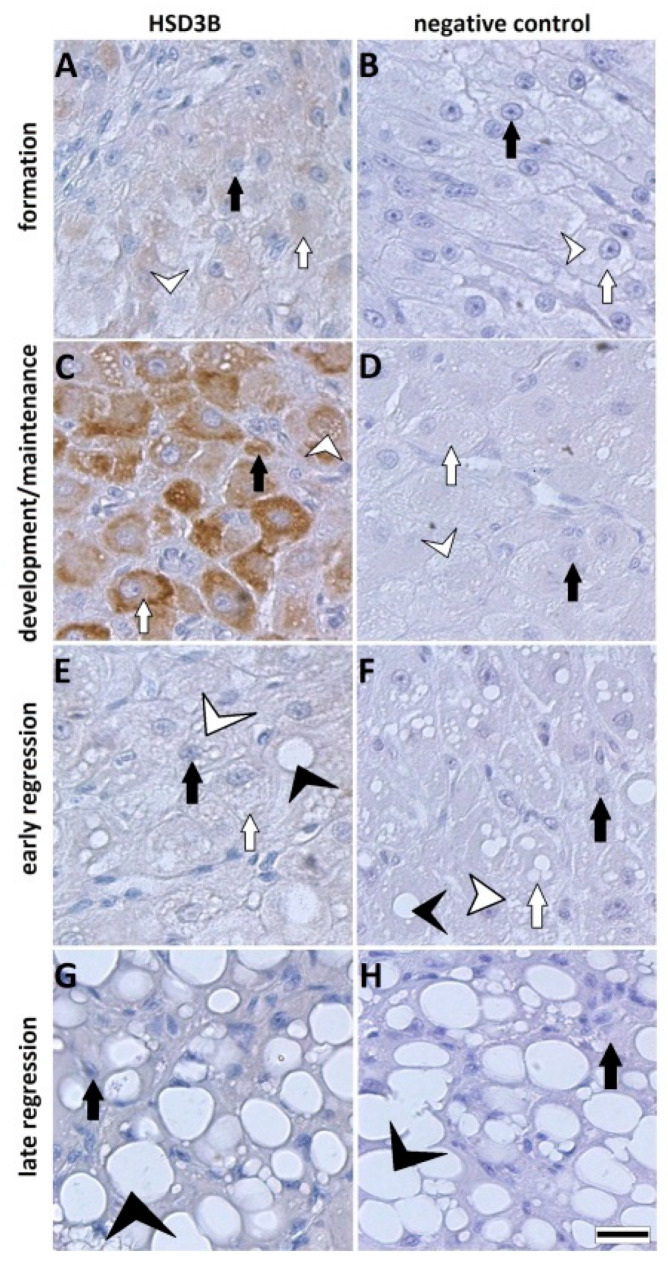
Immunohistochemical staining for HSD3B (**A**,**C**,**E**,**G**) and negative control (**B**,**D**,**F**,**H**) in CL from the domestic cat at different developmental stages. HSD3B protein signal was detected in a cytoplasm of SLC and LLC. Scale bar is equal to 20 µm.

**Figure 5:**
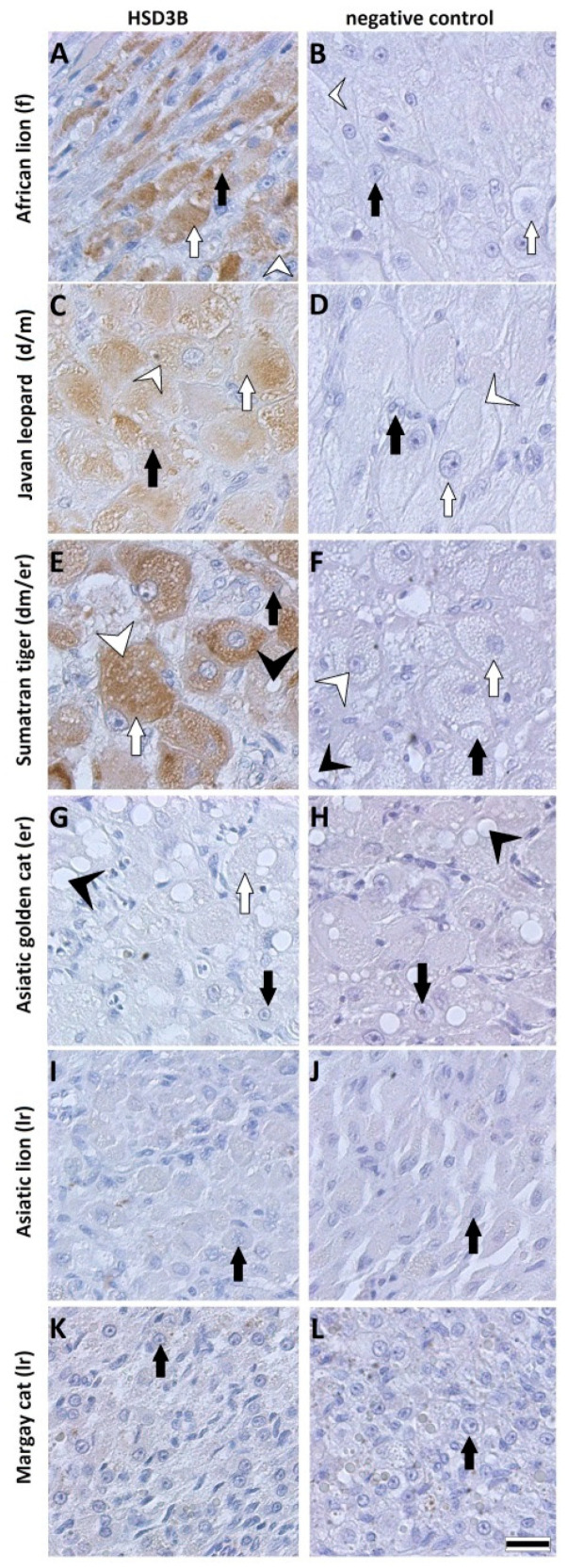
Immunohistochemical staining for HSD3B and negative control in CL from the African lion (**A**,**B**), Javan leopard (**C**,**D**), Sumatran tiger (**E**,**F**), Asiatic golden cat (**G**,**H**) Asiatic lion (**I**,**J**) and Margay cat (**K**,**L**). HSD3B protein signal was detected in a cytoplasm of SLC and LLC. f—formation, dm—development/maintenance, er—early regression, lr—late regression. Scale bar is equal to 20 µm.

By immunohistology, the expression of HSD3B proteins was localized on the tissue sections of CL (Figure 4 and Figure 5). HSD3B protein signals were found in the cytoplasm of large and small luteal cells. In the domestic cat, the protein signal for HSD3B was moderate at the formation stage (Figure 4A) and was strongest at the development/maintenance stage (Figure 4C). Very weak or no signals were observed on the CL when the first signs of structural regression appeared and regression progressed (Figure 4E,G). Signals for the HSD3B protein in CL from wild felids (Figure 5) fit the observations for CL from the domestic cat, where the signal was moderate or strong during the formation and development/maintenance stages and was not detectable or very low during regression stages. Exceptions were the CL from Sumatran tiger, which were classified to the maintenance/development–early regression stage but expressed a high HSD3B protein signal (Figure 5E).

## 4. Discussion

To our knowledge, this is the first report of successful isolation and short-term culture of steroidogenic luteal cells from wild felids accompanied by functional testing for LH reception and HSD3B activity. Previously, steroidogenic luteal cells have been isolated and cultured in domestic cat [20,25,26] and its functional testing was focused on cholesterol and cAMP [26].

The presented results confirmed that in described wild felids, the corpus luteum was composed of two types of steroidogenic cells, namely, small (SLC) and large luteal cells (LLC) (Figure 1 and Figure 2), similarly to the domestic cat [20,25]. As expected, the composition of isolated steroidogenic luteal cells varied depending on species and CL stages (Table 3, Figure 2). We previously described that the enzymatic isolation method we used allowed us to obtain SLC from domestic cat CL almost exclusively (development/maintenance stage) because LLC were fragile and got lost by this method [20]. However, using the same isolation approach on CL from other felids, we obtained a mixture of isolated cells. Up to 16% of the populations were LLC, depending on the species and stage (Table 3). This may be due to species-specific differences towards enzymatic digestion and forces during isolation. The presented results also indicate that the number of isolated LLC was decreasing with the age of CL in wild felids (Table 3). Isolated cells from CL of wild felids during the formation stage were composed of more LLC, and the size of isolated cells steadily decreased with the age of CL. This is in agreement with our unpublished data in domestic cats, where we observed only in isolated cells from the formation stage (enzymatic method) a small number of large luteal cells, whereas they were nearly not present in isolations from later stages. Furthermore, cell size decreased during life cycle in the domestic cat. This is in contrast to results of Arikan et al., who used a different enzymatic method and were able to isolate more LLC from the cell populations of the domestic cat, with higher percentages of LLC in older CL [25]. As LLC of different CL stages could contain two types of vacuolization [8], we suggest that such vacuoles could be cellular structures that limit the isolation of LLC by our enzymatic method in felids.

We used P4 secreted to culture medium to describe the functionality of luteal cells. Previously, we observed a steep drop in hormone content after the first medium change in luteal cell culture [20], suggesting that the hormone decrease reflected the adaptation to culture condition during day 1. The immediate decrease in steroid producing activity after inoculation to the culture disk has been described for rat and bovine luteal cells [27,28]. Regardless, we found tremendous differences in P4 concentration between the species and stages, although the inoculated luteal cell concentration was identical. Very high P4 secretion to cell culture medium was found for the earlier CL stages of wild felids (Figure 3A,C), but for these cell cultures, the decrease in P4 concentration between day 1 and day 2 was greater than 80%, although the total P4 concentration was still at least 10 times higher compared to cultures obtain from regression stages. Interestingly, we did not observe a decrease in P4 for luteal cells of the Asiatic golden cat (Figure 3D). This sample was also interesting because the CL expressed minor morphological signs of regression (Figure 1H), but HSD3B activity and IHC signals were already reduced, confirming functional regression preceding the structural regression [15]. Furthermore, as shown for the domestic cat, luteal cells of the regression stage (Figure 3E) produced around five times less P4 than those from the development/maintenance stage (Figure 3B), which somehow mirror the differences of intraluteal P4 during the CL lifespan [8]. Based on the data for domestic cat and our observation for the culture of luteal cells from wild felid species, we postulate that the very high discrepancies in P4 concentration between the luteal cell cultures from different species presented here were influenced by the different stages of CL. Our results for P4 (Figure 3) were reflected by the steroidogenic enzyme (HSD3B) protein expression (Figure 4 and Figure 5), where this essential enzyme was only clearly detectable during the formation and development/maintenance stages of the CL lifespan. Another factor, which might have had a great impact on P4 secretion, is the composition of the cell cultures. The LLC are known to produce several times more P4 than SLC [20,29,30,31]. Therefore, it is consistent that cell culture from the African lion (CL formation), which has the highest number of LLC, had the highest P4 concentration in the medium (Figure 3A). In addition, mixed cell cultures may be better for both types of steroidogenic luteal cells, mimicking the in vivo conditions and allowing the interactions between both steroidogenic cell types. Previous studies on luteal cells from sheep and pigs showed that SLC and LLC cultured separately produce less progesterone that when they were cultured together [32,33]. The positive impact on in vitro P4 production was also influenced by non-steroidogenic cells in rats [27]. Here, we observed that in mixed cell cultures (wild felids, enzymatic isolation method), LLC behaved differently from LLC of domestic cat (mechanically separated and cultured separately) with regards to morphology [20]. Previously, we showed that LLC mostly do not attach to the bottom of a culture well [20], whereas here, we frequently observed the attachment of LLC (Figure 2). This might indicate better culture condition in cases of mixed cell cultures or an isolation of different LLC subtypes caused by the different isolation methods. It might be suggested that the time between ovariectomy and tissue preparation (transport to the lab) is a limiting factor; however, previous studies showed that storage of ovaries in cooled saline solution inhibited tophonomic changes for 48 h [34]. Our results obtained for the African lion (less than 24 h transport) and the Javan leopard (less than 5 h transport) did not indicate a big difference. Possibly, the large differences in P4 concentrations could also be caused by individual [16] and/or species-specific characteristics. Thongphakdee et al. described that P4 peak concentration in fecal samples of pregnant females can vary up to five times between species [4], but this could be influenced by the number of CL or by different CL sizes too and may not be the result of different P4 expression capacity of luteal cells.

Experiments on different species which investigated the influence of LH on luteal cells used concentrations in a range between 10 and 100 ng/mL [28,35]. For our experiments, using limited and rare materials, we decided to use a concentration of 100 ng/mL, which was previously reported to cause significant changes on estradiol production in cultured granulosa cells from the domestic cat [36]. The used concentration also matched the serum LH peak (over 70 ng/mL) during estrus of the domestic cat [17], but was higher than during the luteal phase of a pregnant queen, in which variable concentrations were measured [16]. Our preliminary experiments on the domestic cat showed that concentrations of 10 ng/mL and 100 ng/mL had almost the same stimulatory effect for P4 production (data not shown), but as we did not have the possibility to test it on samples from wild species, we decided to use 100 ng/mL LH in case cells from the wild felid species require a higher concentration of LH.

We observed LH stimulatory effect for different approaches. Our experiments demonstrated that the almost pure population of SLC (domestic cat) was able to increase P4 production after LH treatment. However, the increase was not as pronounced as described for other species, where P4 concentration in LH treatment group increased several fold in comparison to control group [37]. The luteal cell of wild felids did react to LH (based on measured values, statistical analysis was not performed, *n* = 1), but not in some cultures of cells that were in the regression stage. The stimulatory effect of LH on P4 production of cultured luteal cells was also investigated in other species, e.g., goat [35], pig [38,39,40], sheep [33] and cow [37,41]. Some of these experiments revealed that LH expressed an exclusive or higher stimulatory effect on SLC than on LLC, as a result of a higher number of LHR on small luteal cells. Previously published information about LHR receptor gene expression in SLC and LLC of domestic cat confirmed a different expression on a gene level [20]. At least for felids, we suggest that LLC also respond to LH stimulation.

The decreased activity in expression of HSD3B measured by IHC in the early regression stage of CL is most probably caused by an influence of a luteolytic hormone, of which the action led to functional regression of the gland and shutdown of progesterone production. This result is supported by mRNA analysis for HSD3B in pseudo-pregnant cats, where its expression significantly decreased between the development/maintenance and early regression stage [24].

## 5. Conclusions

In conclusion, our data indicate that the previously established enzymatic isolation method of small luteal cells in the domestic cat can be successfully applied to other felid species, but the ratio of isolated SLC to LLC may be different in comparison to the domestic cat. The results for immunohistochemistry staining revealed that signals of HSD3B decreases after the development/maintenance stage of the gland and indicates functional luteal regression. Obtained results from selected wild felid species were mostly matching to the results of the domestic cat, which is a model species for felids; however, the statistical analysis was not performed for them because of single samples from each species. LH was confirmed to cause a significant increase in P4 production in cultured luteal cells from the development/maintenance stage of domestic cat and caused an increase in P4 concentration in cultured luteal cells from CL of different wild felids at formation and development/maintenance. There was no effect of LH on P4 production in cultured luteal cells of the domestic cat and Asiatic lion CL during the regression stage and a minor stimulatory effect on cells from Asiatic golden cat CL during the early regression stage; therefore, we postulate that the influence of LH, at least in the domestic cat, is dependent on the CL stage. Furthermore, we showed that it is worthwhile to conduct experiments on model species together with single experiments on samples occasionally obtained from rare species. With the limitations of samples from wild felids, this allows to study their functionality of the corpus luteum, or at least a part of it.

## Figures and Tables

**Table 1:** Detailed information about samples from felid species and replicate numbers.

Species	Age of Animal (Years)	Sample Origin	Number of CL Per Animal	Number of Biological Replicates for Cell Cultures	Total Number of Technical Replicates	
					Day 1	Day 2
African lion (*Panthera leo leo*)	7	Givskud Zoo in Denmark	3	1	Control: 6 LH: 14	Control: 3 LH: 11
Domestic cat (*Felis catus*)	*	Tierheim Berlin	*	5 *	Control: 22 LH: 22	Control: 20 LH: 20
				3 *	Control: 18 LH: 18	Control: 9 LH: 9
Javan leopard (*Panthera pardus melas*)	13	Tierpark Berlin	3	1	Control: 6 LH: 14	Control: 3 LH: 11
Sumatran tiger (Panthera tigris sondaica)	18	Zoo Frankfurt	2	**	**	**
Asiatic golden cat (*Catopuma temminckii*)	11	Allwetterzoo Münster	1	1	Control: 6 LH: 9	Control: 3 LH: 6
Asiatic lion (*Panthera leo persica*)	12	Aalborg Zoo in Denmark	undefined	1	Control: 2 LH: 12	Control: 0 *** LH: 10
Margay cat (*Leopardus wiedii*)	10	Tierpark Berlin	1	**	**	**

* Age and number of CL per animal are not shown, as experiments were performed on five different animals in which CLs were classified in the development/maintenance stage and three animals in which CLs were classified in the early regression stage. ** Luteal cell cultures were not performed due to the limited amount of luteal tissue. *** Was not included due to the limited number of isolated cells and design of the study.

**Table 2:** Description of primary and secondary antibodies used for immunohistochemistry.

Antibody	Host	Type	Dilution	Source
anti-HSD3B	mouse	primary	1:500	Santa Cruz Biotechnology Inc., Heidelberg, Germany; sc-100466
ImmPRESS™ VR REAGENT Anti-Mouse IgG Kit	goat	secondary	ready to use	BIOZOL Diagnostica Vertrieb GmbH, Eching, Germany; PEROXIDASECat. No. MP-6402

**Table 3:** Information about the population of isolated cells.

Species	Average Diameter of Isolated Cells ± Standard Deviation (µm)	Number of Counted Cells	Percentage of SLC in Isolated Population of Cells (%)	CL Stage Based on Histology
African lion (*Panthera leo leo*)	14.5 ± 4.6	172	83.7	formation (corpus hemorrhagicum) [f]
Domestic cat (*Felis catus*)	11.5 ± 2.3	253	99.6	development/maintenance [dm]
Javan leopard (*Panthera pardus melas*)	11.5 ± 2.3 * 37.5 ± 5.6 *	100 11	**	development/maintenance [dm]
Asiatic golden cat (*Catopuma temminckii*)	10.5 ± 4.5	136	94.1	regression [er]
Asiatic lion (*Panthera leo persica*)	13.3 ± 4.1	115	93.3	regression [lr]
Margay cat (*Leopardus wiedii*)	10.8 ± 3.1 ***	52	100.0	regression [lr]

* In steroidogenic cells from the Javan leopard, average diameter was calculated separately, because isolated cell suspension contained two distinctive cells types and SLC were in clumps, making counting unreliable. ** Percentage of SLC in the isolated cell population from the Javan leopard was not calculated because SLC were in clumps. *** Steroidogenic cells from the Margay cat were measured after staining them for HSD3B activity.

## Data Availability

The data presented in this study are available on request from the corresponding author.
